# *“I can’t pose a whole heap of questions that I know I don’t have time to follow up”*—Exploring perceptions of an adolescent transition program

**DOI:** 10.1371/journal.pone.0293947

**Published:** 2023-11-13

**Authors:** Kjersti J. Ø. Fløtten, Isabelle Aujoulat, Vegard B. B. Wyller, Anne Lee Solevåg

**Affiliations:** 1 Department of Integrated Care and Health Promotion, Akershus University Hospital, Lørenskog, Norway; 2 Department of Paediatric and Adolescent Medicine, Akershus University Hospital, Lørenskog, Norway; 3 Institute for Clinical Medicine, Faculty of Medicine, University of Oslo, Oslo, Norway; 4 Institute of Health & Society, UCLouvain, Brussels, Belgium; 5 Department of Paediatric and Adolescent Medicine, Oslo University Hospital, Oslo, Norway; UP: University of Pretoria, SOUTH AFRICA

## Abstract

**Background:**

Adolescent transition programs are patient education programs. They are geared towards enabling adolescents with chronic or long-term illnesses to become active partners in their health care and manage their own health. Although there is agreement about their importance, there is not an agreement on content or how they should be delivered. The study reported here was part of the first steps of an action research project.

**Aim:**

Our aim was to explore how health professionals understand the program at our hospital, and their opinions of its implementation. This would advance our knowledge of the practice of the program to support its development.

**Methods:**

We conducted semi-structured individual interviews with 18 physicians and nurses. Data were analysed using qualitative content analysis. In our discussion of the generated data, we use the theory of practice architectures as a lens.

**Results and discussion:**

We generated four themes through the analysis, namely *“*We are (back) at scratch*”*, “Time is always an issue”, “Getting them ready for what is to come–transition as a synonym to transfer” and “Raising topics that go beyond medical issues”. Changes to a practice requires changes to the practice architectures. Practice architectures can both enable and constrain a practice. Our analysis suggests a need for a more unified perception of the program goals, the cultural-discursive arrangements. Health professionals see time as a significant barrier to implementation and changes to the material-economic arrangements are particularly called for, i.e., more time, space and staff to practice the program. These also tie into the social-political arrangements of the program.

**Conclusion:**

There are arrangements in the practice architecture that currently seem to constrain the practice of the program. The practice is currently fragmented both within and across subspecialties. Efforts should be made to establish a more shared understanding of the program among health professionals. Furthermore, we should investigate how the practice of the program can be better supported.

## Introduction

We have seen increased focus in health care on adolescents as a unique group of patients with different needs from the paediatric or adult patient. This is what Alderman *et al*. call: *“[…]a half century–long consequence of the emergence of several significant scientific advances*, *major societal changes relating to teen behaviors*, *and a striking increase in adolescent morbidity and mortality”* [1]. Following this development, attention has risen to the need for transition programs for patients that outgrow the paediatric services [2–4]. In 2003, the Society of Adolescent Medicine stated that the goal of such programs was to optimise health and for each young person to reach their maximum potential [5]. Transition programs are patient education processes for chronically or long-term ill adolescents and should be a series of educational interventions preferably delivered from early adolescence [6]. Internationally these programs focus on adolescents and young adults from the age of 12 to 25. In 2008, Viner argued that better progress was needed in this area and for an increased focus on the ability of adolescents to self-manage their illness [3]. More recent literature reviews demonstrate that there is still a lack of consensus about what the goals and ideal content of transition programs are [7–10].

The program we have at Akershus University Hospital was developed through a participative process over a period of four years [11]. The Department for Paediatric and Adolescent Medicine (DPAM) led the project. The directive was to construct a program that could be adapted and used across diagnoses and subspecialties. When developing the program we found inspiration from programs internationally [12, 13]. Our program has three phases. A preparation phase from 12–16 years of age, where the topic of transition is raised with the adolescent and their parents. This phase focuses on individually adapted information and on the adolescent increasingly taking more responsibility for their own health. The second phase is the transfer phase from 17–18 years of age, where the focus is on getting ready for transfer. The third and final phase is the young adult phase from 19–25, where the adolescent becomes familiar with the adult services [14]. After piloting the program in 2014, the program was adapted and implemented in two to three subspecialties at a time until all had started implementation by the end of 2016.

Development in medicine has led to increasingly sophisticated treatment and more specialised health care. Consequently, health care has been recognised as a complex adaptive system [15]. According to Plsek and Greenhalgh: “A complex adaptive system is a collection of individual agents with freedom to act in ways that are not always totally predictable, and whose actions are interconnected so that one agent’s actions changes the context for other agents” [15]. This complicates development and implementation of interventions as well as evaluations of their effectiveness. Pfadenhauer et al. argue that; “Complex interventions usually comprise multiple components, which may act independently, interdependently, with the ‘active ingredient(s)’ being difficult to specify” [16]. An adolescent transition program can be defined as a complex intervention, involving multiple interacting components and multiple organisational levels [17, 18]. We saw the need to focus on continuous quality improvement of our program and discussed multiple approaches that would be suitable to trail this type of intervention. One was through a project with a qualitative approach. The initial intention of the qualitative project was to investigate stakeholder perceptions of the implementation within paediatrics and furthermore to explore how strengthened health literacy was of value for adolescent empowerment. Action research was chosen as an approach [19]. We framed the project within the five steps of action research described by Kemmis et al. [20]. The project was given the working title *H**ealth L**I**teracy* *F**or young* *I**ndividuals*, *its* *V**alue for* *E**mpowerment and successful transition* (HI-FIVE).

Parallel to the process of developing HI-FIVE, in 2018, DPAM distributed a user satisfaction survey targeting both parents and adolescents. Some of the survey questions were directly related to transition. Although the number of adolescent respondents was low (n = 32), they provided us with valuable feedback:

- 54.8% of the responding adolescents did not know the name of the health professional with main responsibility for their care;
- 46% said they were offered no, little or some option to consult with the physician or other members of their health care team without their parents/guardians present;
- 54.4% said they received no, little or some information about transition to adult care within the last 12 months;
- 63.6% said they did not feel prepared for transfer;
- 40.6% said they had not been offered to join a patient education course that thematise living with a long term or chronic illness.

These results left us with a number of questions. Was the program not practiced to the extent it should be? Alternatively, did the program content lack relevance and miss the target of making adolescents feel empowered? These results fuelled the discussions we had concerning the first steps to take in HI-FIVE.

The study reported in this paper was part of the first phases of HI-FIVE. These are the phases in action research where we investigated how things work in the program in order to discuss the results and make adjustments [20]. Proponents of action research argue that participants in action research begin their projects by asking themselves what they are doing, why they do it and what they hope to achieve [20–22]. According to McNiff, action research starts precisely by asking oneself questions such as: “Is my/our work going as we wish? How do we improve it where necessary?” [23]. Our goal was not to evaluate program fidelity, but to explore how health professionals defined the program and how they describe their practice. Mahon *et al*. argue: *“In practices*, *particular kinds of relevant sayings*, *doings*, *and relatings are harnessed together in some kind of coherent way in the pursuit of the project of the practice”* [24]. We began by asking ourselves what frames, or sayings, are provided to guide present practices [20]. This investigation began with an analysis of the written program material. The analysis made us question whether the program sufficiently addresses all aspects of health [25]. Paired with the results from the user satisfaction survey, the analysis made us wonder whether the program theory and intentions are explicit and shared among stakeholders. In addition, there was the question of how the program was practiced, i.e., the *doings* of the program [20].

The aim of this study was to explore how health professionals who run the transition program understand the program. Secondly, to explore their opinions about implementation and factors that influence implementation. This would enhance our knowledge of the practice of the program and provide us with an opportunity to amend the program as needed.

## Methodology

### Context

The site of our study was the DPAM at a large Norwegian hospital. The department sees patients 0–18 year of age and houses most paediatric subspecialties within somatic care in a Norwegian context. Apart from a Child and Adolescent Mental Health Services (CAMHS) team, mental health services are provided in a separate department. The hospital was amongst the first in Norway to raise the age limit from 14 to 18 years for patient treated in DPAM in 2008 and the first to develop a cross diagnostic transition program. In 2018, the Commissioner’s Document from the four Regional Health Trusts to all Norwegian hospitals stated that they should establish routines for adolescent transition. In a 2019 survey by the Norwegian Society of Paediatricians, 16 of the 19 responding said they had routines in place, but few stated their routines covered all subspecialties [26]. Physicians and nurses in the outpatient clinic have the main responsibility for the program.

### Study design

We performed semi-structured individual interviews [27]. An interview guide was developed by the first and last authors in collaboration with the head of the outpatient clinic and the head of the CAMHS team. We intended to use the guide flexibly, leaving room for the conversation to develop spontaneously. This allows the participants to raise topics not covered by the interview guide [27]. We see interviews as a co-construction between the researcher and the participant [27–29].

### Recruitment and data generation

Recruitment was done with the help of key persons in the clinic, amongst others the last author. Our aim was to explore a variety of experiences and opinions of the program. Inclusion criteria were: 1) physician or nurse in the outpatient clinic and, 2) that were both experienced and less experienced with the program. We aimed to recruit participants from all subspecialties.

The first author conducted all interviews. They were audio recorded in all but two cases. In these two cases field notes were written. Eighteen interviews (11 nurses and 7 physicians) were undertaken in spring/summer 2018, representing all subspecialties and reaching saturation in the topics that were being raised [27]. The interviews lasted between 30 minutes and 1 hour and 15 minutes.

### Data analysis

The first author transcribed the recorded interviews and field notes from the two unrecorded interviews. These documents made up the *units of analysis* in a qualitative content analysis as outlined by Graneheim and Lundman [29]. This form of analysis is suitable because: *“It focuses on subject and context and emphasizes variation*, *e*.*g*., *similarities within and differences between parts of the text”* [30]. We acknowledge the multiple meanings of a text and that the analysis we present is one story [29].

The first and last author read the documents multiple times inductively to get a sense of the whole, focusing on how health professionals talk about the program. The two authors discussed initial ideas. The text was then divided into *meaning units* by the first author [29]. The meaning units were condensed, meaning that they were shortened without taking away the essence [29]. Meaning units found relevant for the research questions were highlighted and entered into one document. The document was imported to and coded in NVIVO (QSR International, Melbourne, Australia). The first author performed the initial coding. The first and the last author discussed the coding. This involved a back and forth movement between the codes, meaning units and the full text. Initial themes were developed [30], translated and draft descriptions were made. These were revised and finalised after input on the draft from all co-authors.

### Ethics

Information about the project was given in writing and orally prior to interviews. Written informed consent was obtained. Participants were informed that participation was voluntary and that consent could be withdrawn at any time. Consent to record the interview was obtained. Each interview was coded with a number. No information that could identify the participant was recorded in the transcripts. Only the first author had access to the audio recordings and the list of codes.

The project protocol was submitted to the Regional Committee for Health Research Ethics (#2017/2313 C), who considered the project to be exempt from their review due to the research focus being on health services and not on generating knowledge about illness or health. The Data Protection Officer (#2017_211) at the hospital approved the project.

The topics to be discussed in the interviews were not perceived as sensitive, but we remained conscious that participants could have a different perception of this than us [27].

## Results

We generated four themes through our analysis. They are presented in the following paragraphs, supported by quotes. We do not differentiate between the two professions in the reporting because the topics brought up in the conversations overlap to such an extent that we see no need to do so. Furthermore, the two groups share the main responsibility for the program.

### We are (back) at scratch

Our analysis paints a picture of great differences when it comes to how well implemented health professionals perceive the program to be. A substantial proportion define themselves still ‘(back) at scratch’. Some are having only introductory conversations about how to run the program.

In [subspecialty] we do not have a transition program running. But we have started talking about it.

When initially describing the program, health professionals refer to the use of checklists and program material in conversations with adolescents, having parts of the consultation without parents present and about themselves collaborating closer with the adult receiving department. Some subspecialties are perceived to be model implementers and some argue that others have never really managed to follow suit.

The ones who have been particularly involved in transition are [specialists]. They have sort of been champions for it. It was in a way natural that they piloted [the program]. However, I feel that others did not really follow them. At least not in the field I work in.

Some say that they did get a system up and running, but that for different reasons, most commonly due to turnover of key personnel, this system has collapsed. Participants who are new to the team and therefore define themselves as new to the program say they would have wanted more information about what their predecessor actually did when they delivered the program. However, knowledge about other topics and procedures was prioritised in the, often short, periods of training. Participants argue that for the program to run successfully there is a need for champions in *both* professions.

Lack of implementation does not equal lack of relevance. In general, the participants describe a positive attitude towards the program in the clinic and among colleagues:

Most people are positive. There are few reasons to… or, it is hard to find arguments for it being terribly wrong to make children able to become adults. It would be arguing against common sense to say you do not believe in it.

Most speak of being at a different level of awareness about adolescent medicine and transition than before the program was launched. However, they admit that transition might not be the most common topic of conversation over lunch:

I do not think there has been many lunch discussions about transition. […] I think that what one talks about from the outpatient clinic is the more rare clinical cases, not; that was a really good example of transition.

There is a desire for more opportunities to discuss and exchange ideas about the program and to learn from each other. Those who belong to subspecialists perceived as model implementers also voice a need for more knowledge exchange and discussions. They too see flaws in their practice.

Nevertheless, the participants perceive that a transition program and the focus on adolescent medicine has become lasting. Reasons for not having come further in implementation revolve mainly around two topics. First, the number of adolescents seen by the subspecialty:

It is so much rarer within the field that I work, in comparison to others here at the outpatient clinic, that [we have someone who] will be transferred to adult services.

The health professionals acknowledge that this is not an excuse for lack of focus on transition, but state that practice does make you better. The second main reason is caring for adolescents who will not transfer to one adult department. It is argued that some subspecialties have it easier, because their adolescents are all more or less walking along the same road to one clearly defined recipient. Because health professionals see their collaboration with the receiving adult service as highly central to the program, having no clear receiving service or having multiple receiving services in their opinion complicates matters.

### Time is always an issue

Time is a recurring topic in our analysis. First, time is problematized when it comes to *the amount of time allocated for a consultation* and what topics one is able to raise in a very short amount of time.

When you are in a hurry, you are a bit afraid of opening up for things that you do not have time to follow up. So sometimes, you are a bit closed off [in your communication] on purpose.

This concerns having time for a conversation on broader topics in that exact consultation, but also the potential time required to follow up topics raised by the adolescents:

If you pose a question about something and you are preoccupied with something, to be trustworthy you have to set aside time to solve the problem that you ask the adolescent to present to you. I can’t pose a whole heap of questions that I know I do not have time to follow up.

In other words, health professionals believe that if they had more time for each consultation they would be able to address more topics with the adolescents. Sometimes this refers to covering more topics from the program material. Other times it refers to covering topics they find to go beyond the material:

What I think the transition program should have more of, both at the end and in the beginning. [Is] that one should focus not just on the purely medical [aspects], but also on being an adolescent.

Yes. I do wonder how people are doing. I do. My problem is that I never have enough time to ask about it. The next patient is outside waiting. I wish I had the opportunity to. I am not afraid of talking about it.

There is a perception among the health professionals of a lack of time. A minority of the subspecialties have separate transition consultations. Most have to fit conversations about transition into an already packed consultation. Getting through the medical aspects thus takes priority. In other instances, time *during* consultations is problematized when it comes to being able to be more systematic so that all get an equal offer of the program. Yet, we found examples of subspecialties that have ‘made’ time without further ado. They schedule longer consultations for adolescents on a regular basis.

The third issue to do with time is the *experience of being too short of time in general* to be able to implement the program:

But [I] experience it a bit like a lot of other things in a hospital where you have grand plans, and you put them into motion, and then you are done with it. Without having set aside extra time or us getting any resources or anything at all to enable us in the daily running. It is just another task that is to be done in the same amount of time as before. So I do not find it to be facilitated.

In other words, time also ties into other resources such as number of designated staff, because it means enough time divided between enough people to follow through properly. Moreover, time is an issue when it comes to allocation of time in rooms that are appropriate for having conversations in private.

The fourth aspect of time involved discussions around what is *the right time to start the program* for the adolescents. The guideline states that the first phase of the program should start from around the age of 12. Some state that they have made adjustments from the original timeline.

When we started running the transition program it was meant to be from age 14. I experience that in practice it is too early. That they do not get too much out of it and find it a bit scary. So, we’ve waited till they’re 16 and then they have time alone with me first.

Others state the opposite. That they currently do not start early enough and should have a goal to get started at the age of 12 as the guideline outlines.

### Getting them ready for what is to come—transition as a synonym to transfer

There is a general agreement that a main goal of the program is getting the adolescents prepared for what is to come in adult services:

I think that amongst other things it is to ensure continuity from where the adolescent has received care in paediatrics to the adult department. And, of course to prepare the adolescent for what comes. There are quite large differences between paediatrics and adult care regardless of subspecialty.

Health professionals see this as a strength of the program. Some also talk about ‘transition’ as synonymous with ‘being transferred’:

I had almost never heard of transition before, in terms of transfer to the adult services.

Knowledge and skills regarding their diagnosis and treatment are competences that are seen as central for the adolescents to be able to fend for themselves after being transferred and to make the transfer less of a shock. Medical management and knowledge about the diagnosis, how to book an appointment, and reorder prescriptions are emphasized by the participants. Furthermore, the participants stress the importance of making adolescents aware that these tasks are now increasingly their own responsibility.

It is most important to make them cognisant. Cognisant of what their illness entails. Potential limitations, possibilities and cognisant of medication. The importance of taking them and how they work. That they learn why they need their medicine to stay well. And making them responsible, step-by-step.

Aiding the adolescent to take over control of disease management and helping the parents to let go is a frequently raised topic.

### Raising topics that go beyond medical issues

Our participants talked about topics they perceive to go ‘beyond medical management’. Frequently, they label these as ‘adolescence-specific topics’. This label refers to topics such as sex, alcohol, tattoos and further studies or vocational training. Some say that they frequently get questions about these topics. Others find the topics important to raise unprompted because these areas of life affect or are affected by the chronic condition of the adolescent.

I think they find it important that we, that doesn’t really relate to the checklist though, but that we ask them about other things in life. That we are interested in them. Not just diseases and numbers. That is important. The checklist doesn’t really say too much about that.

Topics that go beyond medical management are also topics that revolve around coping with an illness. Participants who raise this topic talk of a close collaboration with the CAMHS team. Others feel that this is a topic that is not raised enough.

So that touches on coping strategies. How to live with [symptom]. If you cannot get rid of it completely, how do you live with it? We do have very little focus on that. On how to live with something. How to not get a hundred percent well but manage to live with things.

Mainly two reasons are given for not bringing up these topics. One ties into the theme of time as they revolve around lacking the time to discuss topics beyond the medical. The second revolves around feeling one lacks knowledge and skills to be able to discuss such topics:

There are probably some things that we could have worked on here as well. And some things we do. But often times I feel that I lack tools to land things with them here and put it to rest.

In other words, some health professionals feel that they need a different set of skills to be able to raise certain topics with the adolescents.

## Discussion

We found the recurring threads of meaning [29] in our analysis to be: *‘*We are (back) at scratch*’*, *‘*Time is always an issue*’*, *‘*Getting them ready for what is to come–transition as a synonym to transfer*’* and *‘*Raising topics that go beyond medical issues*’*. We asked ourselves ‘what is going on here?’ [20] and first of all our analysis told us that the program was not practiced consistently in all subspecialties. In addition, the perception of the program goals varied between the participants from a focus on medical management and transfer to a broader focus on topics beyond medical management. To us, a timely question then became ‘how did this come to be?’ [20]. What structures are present, or not, that shape the practice?

Practice theories are relevant when exploring transition programs and with them the recognition that; “practices are situated, social and relational” [24]. In the following discussion, we use the theory of practice architectures as an analytical tool in an attempt to advance our understanding of our results [20, 24]. We find using this theory useful because: *“Practice theory provides lenses which makes examination of practices possible*, *and in doing so enables useful accounts of how practice happen*, *how they are mediated*, *and their role in the constitution of social life”*[24]. According to the theory of practice architectures, practice architectures are what holds a practice in place [20]. They are made up of three different forms of arrangements, namely *cultural-discursive*, *material-economic* and *social*–*political* arrangements. According to Kemmis *et al*. changes to a practice thus requires more than actors acquiring new knowledge [20]. It also requires changes to the conditions under which one practices [20].

According to Mahon *et al*. the cultural-discursive arrangements in a practice: “[…]*can constrain and/or enable what it is relevant and appropriate to say (and think) in performing*, *describing*, *interpreting*, *or justifying the practice”* [24]. In our context, this would refer to the language and discourse used in and about the transition program by health professionals, by managers or by adolescents and parents. These are the program *sayings*, and they constrain or enable what is seen as relevant for the program both explicitly as in a shared language but also tacitly. The results from our analysis that show differences in the understanding of the programs goals fall under these arrangements. Paired with the results from our earlier analysis of the program material [25] we question whether these arrangements have been adequately addressed and whether they at present constrain or enable the practice. Campbell *et al*. argue that:*“For an intervention to have a credible chance of improving health or health care*, *there must be a clear description of the problem and a clear understanding of how the intervention is likely to work”* [31]. Are we in agreement about what we think the problem to be solved is and thus how the program is likely to work? If how we talk about the program continuous to revolve around transfer, about what happens after transfer or about knowledge of medical management, other broader definitions of health will tacitly be downplayed.

The *material-economic* arrangements of a practice are resources that make possible or shape the *doings* in a practice. They are resources in physical space, human resources or financial resources [32]. Our results highlight time as scarce resource in multiple ways. Time for consultations, enough staff and time allocated in consultation rooms (i.e. physical space) are aspects that belong within these arrangements. Furthermore, we question what is present or not in the practice architecture when some subspecialties make changes to the material-economic arrangements by adding extra time for consultations, while others perceive that they do not have the opportunity to do so. This last point ties into the *social-political* arrangements, or *relatings* of the practice. It seems that not all health professionals feel that they have a mandate to make adjustments in the material-economic arrangements. This not only ties in to the social-political arrangements locally at our hospital but into the larger national debate on efficiency in health care.

Through our analysis, we came to realise that even though our program is cross diagnostic, each subspecialty should be seen as a context of its own. According to the theory of practice architectures the site holds the practice in place [20]. One could question what the site of the program really is. Is it DPAM as a whole? Is it the outpatient clinic? Or is it each subspecialty? This would mean that we are in fact looking at a multi context complex intervention. The question we raise is whether this has been sufficiently acknowledged when considering the need for implementation support.

### Methodological considerations

A text has multiple meanings [28, 29] and we acknowledge that our analysis is only one story. We have tried to enhance trustworthiness through a detailed description of the steps taken to generate and analyse our data. Our pre-understandings may represent both strengths and limitations [28]. They are limitations if one is not conscious about them and thus not open to alternative ways of interpretation, strengths if we use them as assets in the interpretation and discussion of the data. The research team comprised of people with varied backgrounds who have experience from the daily life in the clinic, experience from health promotion and the use of qualitative methodology. We find this to be a strength because it added different perspectives to our discussions about the data.

The results must be understood in light of the context(s) in which the interviews were performed. It is a strength of the study that all subspecialties and the two professions with main responsibility for the program are represented. We believe that this gave us a more complex picture.

## Conclusion

Changes to a practice require changes to the practice architectures. Our analysis suggests a need for a more unified perception of the program goals, the *cultural-discursive* arrangements. Efforts should be made to establish a more common understanding of what the main goals of the program are. This may be done through opening up spaces for discussions and exchange of thoughts about and experiences with the program. Each subspecialty should establish meeting places for such exchanges. Furthermore, we see a need for adjustments of how transition consultations are organised. Factors that influence implementation are strongly associated with time, more specifically, a lack thereof. This implies that changes to the *material-economic* arrangement are particularly called for, i.e., more time, space and staff to practice the transition program.
